# A Nanoscale Low-Power Resistorless Voltage Reference with High PSRR

**DOI:** 10.1186/s11671-019-2864-7

**Published:** 2019-01-24

**Authors:** Zekun Zhou, Jianwen Cao, Yunkun Wang, Yue Shi, Zhuo Wang, Bo Zhang

**Affiliations:** 1 State Key Laboratory of Electronic Thin Films and Integrated Devices, University of Electronic Science and Technology of China, Chengdu, 610054 Sichuan China; 2 College of Communication Engineering, Chengdu University of Information Technology, Chengdu, 610225 China

**Keywords:** Subthreshold, Low-power, High PSRR, Resistorless, Nanoscale

## Abstract

In this paper, a nano-watt resistorless subthreshold voltage reference with high-power supply rejection ratio (PSRR) is presented. A self-biased MOS voltage divider is proposed to provide bias current for whole voltage reference, which is a positive temperature coefficient (TC) current containing threshold voltage characteristics. By injecting the generated current into a transistor with a different threshold voltage, a delta threshold voltage with a greatly reduced negative TC is realized and temperature-compensated by a generated positive TC item at the same time. Therefore, a temperature-stable voltage reference is achieved in the proposed compacted method with low power consumption and high PSRR. Verification results with 65-nm CMOS technology demonstrate that the minimum supply voltage can be as low as 0.35 V with a 0.00182-mm^2^ active area. The generated reference voltage is 148 mV, with a TC of 28 ppm/°C for the − 30 to 80 °C temperature range. The line sensitivity is 1.8 mV/V, and the PSRR without any filtering capacitor at 100 Hz is 53 dB with a 2.28-nW power consumption.

## Introduction

Voltage reference is one of the core modules in electronic systems, which is widely used in medical electronics, power managements, wireless environmental sensors, and communication circuits. As the supply voltage of electronic systems continues to decrease with technology improvement, the requirements for a low-power voltage reference with nanoscale technology are critically increasing [1, 2].

Conventional voltage references are based on a bandgap reference (BGR) circuit, which is a weighted sum of *V*_BE_ and thermal voltage [3, 4]. However, due to the nonlinear temperature behavior of *V*_BE_, it is essential to use curvature compensation approaches to improve the precision of BGR [5, 6]. Another disadvantage of BGR is the power consumption. The *V*_BE_ is around 0.7 V without shrinking down with process improvement, which absolutely restricts the supply voltage. These make BGRs unsuitable for low-voltage and nanoscale applications.

In order to achieve low-power operation, MOS-only subthreshold voltage references are gradually adopted [7–10]. As transistors in a weak inversion region have inherent advantages in low-power applications with quite small current, the power consumption of relative voltage references can be effectively reduced. Besides, since the characteristics of metal-oxide-semiconductor field-effect transistor (MOSFET) are consistent with process improvement, voltage reference based on MOSFET is more adaptable to advanced technologies. In addition, the usage of resistors should also be avoided in low-power applications. Since the current in the voltage reference is usually inversely proportional to resistance value, low-power dissipation means high-ohmic resistors [10], which can induce large noise occupying a large chip area.

Power supply rejection ratio (PSRR) is another important parameter of voltage reference. Conventional solutions to improve PSRR are at the cost of chip area and power consumption, such as additional amplifiers [11], long channel transistors [12], cascode structures, and additional gain stage [13].

In order to overcome the mentioned issues above, a nano-watt MOSFET-based resistorless subthreshold voltage reference with high PSRR is proposed in this brief, which is suitable for advanced technology, such as nanoscale process. A self-biased MOSFET voltage divider for PSRR enhancement is adopted in the proposed voltage reference, which can generate a positive temperature coefficient (TC) current containing threshold voltage characteristics. The current serves as bias currents for the whole voltage reference. Besides, the threshold voltage embedded in the bias current is reproduced by injecting bias current into MOSFET with different threshold voltages in the paper. With the proposed method, a delta threshold voltage (*∆V*_TH_) with greatly reduced negative TC is obtained. Besides, a weighted proportional to absolute temperature (PTAT) item is also obtained, while a weighted sum of *∆V*_TH_ and PTAT voltage is realized at the same time. Due to the mutual TC cancelation of two different threshold voltages, the required PTAT voltage can be greatly reduced for temperature compensation. By this method, a MOSFET-only resistorless voltage reference is achieved by a compacted structure with low power consumption.

## Method

**Fig. 1:**
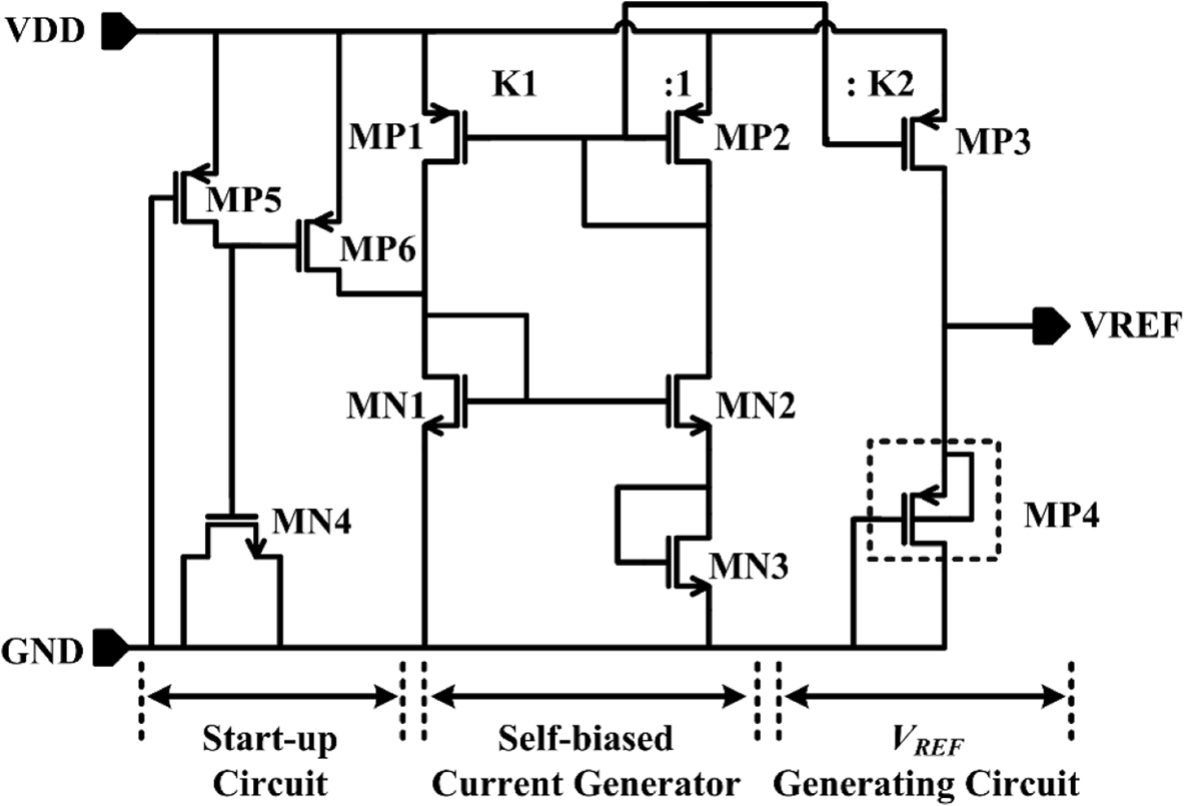
Schematic of proposed voltage reference

As shown in Fig. 1, the proposed voltage reference is composed of a start-up circuit, a self-biased current generator, and a *V*_REF_ generating circuit. All the n-channel MOSFETs are a medium threshold voltage N-type metal-oxide-semiconductor (mvt NMOS). MP4 is a high threshold voltage transistor P-type metal-oxide-semiconductor (hvt PMOS), and the other p-channel MOSFETs are a medium threshold voltage PMOS (mvt PMOS). All the transistors shown in Fig. 1 operate in the subthreshold region, except those in the start-up circuit.

### Start-Up Circuit

The start-up circuit consists of MP5, MP6, and MN4. At the beginning of a power-on stage, the gate potential of MP6 is low and MP6 is turned on. The current generated by MP6 makes the gate potential of MN1 and MN2 rise, and the whole circuit starts to work. At the same time, MP5 charges the start-up capacitor, MN4. With the charging procedure of MN4, transistor MP6 is gradually turned off, which makes the start-up circuit to be broken away from the core of the proposed voltage reference without additional power dissipation. By this method, the proposed voltage reference can work in a desired operating point while avoiding a degeneration point.

### Self-Biased Current Generator

The middle part in Fig. 1 is a self-biased current generator, which is based on a MOSFET-only voltage divider. The bias current with positive TC for the whole voltage reference is generated in this part, which is relevant to the medium threshold voltage of NMOS. The unique characteristic of the presented bias current is adopted to realize the proposed voltage reference in a convenient way, which will be analyzed in the “Method” section.

With regard to voltage current characteristic of a transistor in the subthreshold region, the drain current of the transistor in the subthreshold becomes almost independent of *V*_DS_ with *V*_DS_> 4*V*_T_, where *V*_T_ *= kT/q* is the thermal voltage, *k* is the Boltzmann constant, *q* is the elementary charge, and *T* is the absolute temperature. Hence, the current can be expressed as:

1 $$ {I}_{\mathrm{D}}={SI}_{\mathrm{SQ}}\exp \left(\frac{V_{\mathrm{GS}}-{V}_{\mathrm{T}\mathrm{H}}}{mV_{\mathrm{T}}}\right) $$

where *S = W/L* is the aspect ratio, *m* is the subthreshold slope factor, *V*_TH_ is the threshold voltage, and *I*_SQ_ represents the specific current and is presented by:

2 $$ {I}_{\mathrm{SQ}}=\mu {C}_{\mathrm{OX}}\left(m-1\right){V_T}^2 $$

where *μ* is the carrier mobility and *C*_OX_ is the oxide capacitance per unit area.

Therefore, the currents through MOSFET-only voltage divider, formed by MN1, MN2, and MN3, can be expressed as follows:

3 $$ {I}_{\mathrm{D}\_\mathrm{MN}1}={S}_{\mathrm{MN}1}{I}_{\mathrm{SQN}}\exp \left(\frac{V_{\mathrm{GS}\_\mathrm{MN}1}-{V}_{\mathrm{T}\mathrm{HN}}}{mV_{\mathrm{T}}}\right) $$

4 $$ {I}_{\mathrm{D}\_\mathrm{MN}2}={S}_{\mathrm{MN}2}{I}_{\mathrm{SQN}}\exp \left(\frac{V_{\mathrm{GS}\_\mathrm{MN}2}-{V}_{\mathrm{T}\mathrm{HN}}}{mV_{\mathrm{T}}}\right) $$

5 $$ {I}_{\mathrm{D}\_\mathrm{MN}3}={S}_{\mathrm{MN}3}{I}_{\mathrm{SQN}}\exp \left(\frac{V_{\mathrm{GS}\_\mathrm{MN}3}-{V}_{\mathrm{T}\mathrm{HN}}}{mV_{\mathrm{T}}}\right) $$

where *I*_SQN_ is the specific current of NMOS and *V*_THN_ is the threshold voltage of NMOS.

Since the aspect ratios of MN2 and MN3 are the same and *I*_D_MN2_ *= I*_D_MN3_, *V*_GS_MN2_ = *V*_GS_MN3_ is guaranteed. This makes *V*_GS_MN1_ = 2*V*_GS_MN2_. Besides, the PMOS transistors form the current mirrors and define the current ratios *K*_1_ *= S*_MP1_*/S*_MP2_ and *K*_2_ *= S*_MP3_*/S*_MP2_. The relationship of drain currents between MN1 and MN2 can be expressed as:

6 $$ {I}_{\mathrm{D}\_\mathrm{MN}1}={K}_1{I}_{\mathrm{D}\_\mathrm{MN}2} $$

Combined with Eqs. (3)–(6), the *V*_GS_MN2_ and *I*_D_MN2_ can be given by:

7 $$ {V}_{\mathrm{GS}\_\mathrm{MN}2}={mV}_{\mathrm{T}}\ln \left(\frac{K_1{S}_{\mathrm{MN}2}}{S_{\mathrm{MN}1}}\right) $$

8 $$ {I}_{\mathrm{D}\_\mathrm{MN}2}={S}_{\mathrm{MN}2}{I}_{\mathrm{SQN}}\exp \left(\ln \frac{K_1{S}_{\mathrm{MN}2}}{S_{\mathrm{MN}1}}-\frac{V_{\mathrm{T}\mathrm{HN}}}{mV_{\mathrm{T}}}\right) $$

For the convenience of analysis, Eq. (8) can be abbreviated as:

9 $$ {I}_{\mathrm{D}\_\mathrm{MN}2}={aT}^{2-{n}_1}\exp \left(b-\frac{V_{\mathrm{T}\mathrm{HN}}}{mV_{\mathrm{T}}}\right) $$

where *a = S*_MN2_*μ*_*n*0_*C*_OX_(*m −* 1)(*k/q*)^2^ and *b =* ln(*K*_1_*S*_MN2_*/S*_MN1_) are independent of temperature, *μ*_*n*0_ is a temperature-independent factor of carrier mobility, and *n*_1_ is the absolute temperature exponent term of carrier mobility, which is usually around 1.5.

As shown in Eq. (9), threshold voltage *V*_THN_ is complementary to absolute temperature (CTAT), while thermal voltage *V*_T_ is proportional to absolute temperature (PTAT). As the temperature increases, *V*_THN_/(*mV*_T_) will reduce, so that the positive current characteristics of the bias current will be enhanced.

By this method, a positive TC bias current is achieved by MOSFET-only structure, which carries the characteristics of NMOS threshold voltage.

### *V*_REF_ Generating Circuit

The *V*_REF_ generating circuit is shown in the right part of Fig. 1, which is only formed by two transistors, MP3 and MP4. Due to the subthreshold region operation, *I*_D_MP4_ can be written as:

10 $$ {I}_{\mathrm{D}\_\mathrm{MP}4}={S}_{\mathrm{MN}4}{I}_{\mathrm{SQP}}\exp \left(\frac{\mid {V}_{\mathrm{GS}\_\mathrm{MP}4}\mid -\mid {V}_{\mathrm{T}\mathrm{HP}}\mid }{mV_{\mathrm{T}}}\right) $$

where *I*_SQP_ is the specific current of PMOS and *V*_THP_ is the *V*_TH_ of PMOS.

Since *I*_D_MP4_ *= K*_2_*I*_D_MN2_, the characteristics of NMOS threshold voltage, *V*_THN_, can be transferred to the output node and be superposed with the characteristics of PMOS threshold voltage, *V*_THP_. From Eqs. (8) and (10), *V*_REF_ can be written as:

11 $$ {V}_{\mathrm{REF}}=\mid {V}_{\mathrm{T}\mathrm{HP}}\mid -{V}_{\mathrm{T}\mathrm{HN}}+{mV}_{\mathrm{T}}\ln \left(\frac{K_2{S}_{\mathrm{MN}2}{I}_{\mathrm{SQn}}}{S_{\mathrm{MP}4}{I}_{\mathrm{SQP}}}\right)+{mV}_{\mathrm{T}}\ln \left(\frac{K_1{S}_{\mathrm{MN}2}}{S_{\mathrm{MN}1}}\right) $$

As shown in the first two items of Eq. (11), a delta threshold voltage is realized. Since *V*_TH_ = *V*_TH0_ − *βT*, where *V*_TH0_ is the threshold voltage at 0 K and *β* is the TC of the threshold voltage, the generated delta threshold voltage is a complementary to the absolute temperature (CTAT) voltage with greatly shrunken TC with |*βV*_THP_| > *βV*_THN_. Besides, two additional PTAT voltages are simultaneously realized and shown in the last two items of Eq. (11), which are adopted to cancel the reduced TC of delta threshold voltage. Therefore, a compacted temperature-stable reference voltage is achieved without a complicated structure, which is stable at |*V*_THP0_| − *V*_THN0_.

Based on the previous analysis, a low-power MOSFET-only voltage reference is realized in this paper which only requires three branches in the core. With the unique characteristics of a self-biased current source, one diode-connected PMOS is adopted to achieve a CTAT voltage with shrunken TC, PTAT voltage generator, and weighted summation at the same time. What is more, the proposed structure is only constructed by MOSFETs, and the generated reference voltage is proportional to the delta threshold voltage. Therefore, the proposed voltage reference is more suitable for low power consumption applications with nanoscale technology, which can be further extended to more advanced technologies.

### PSRR of Proposed Voltage Reference

**Fig. 2:**
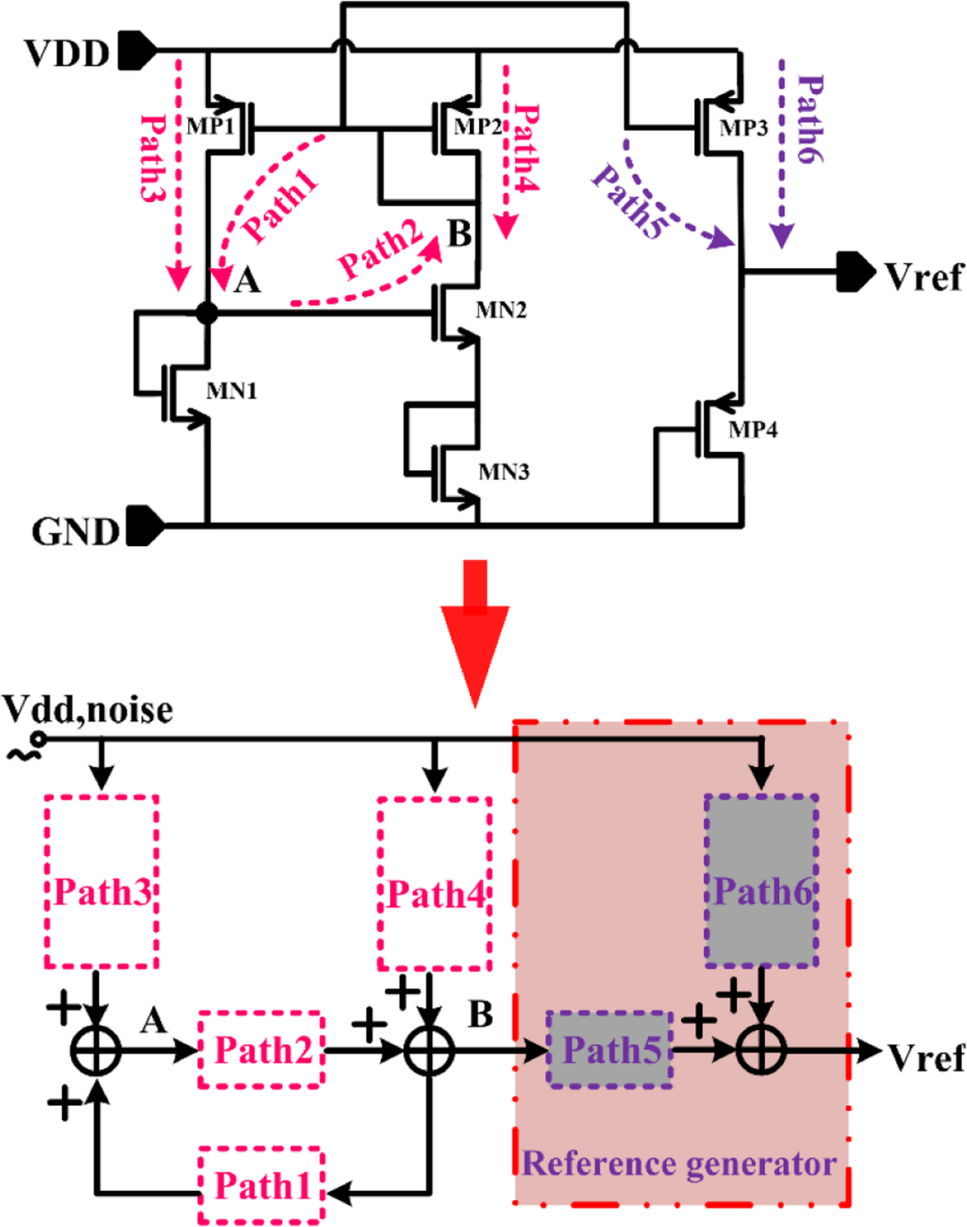
The paths of supply voltage noise

In order to illustrate the PSRR performance, the paths from supply voltage noise to *V*_REF_ and corresponding equivalent function diagrams are shown in Fig. 2.

**Fig. 3:**
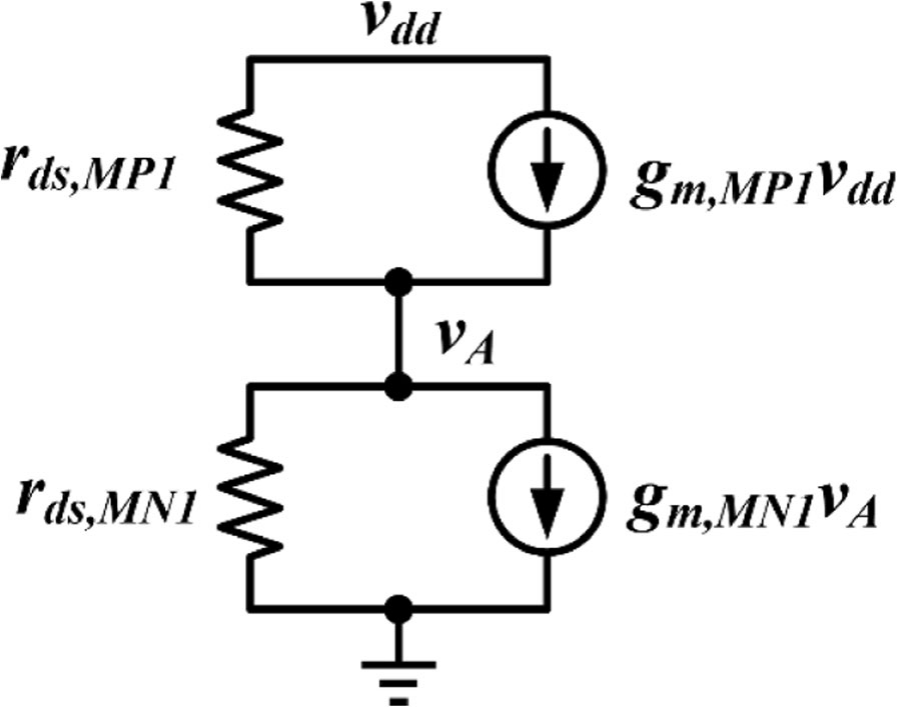
Small signal model of path 3

Based on Fig. 2, the small-signal model of path 3 is shown in Fig. 3, and the following equation can be obtained:

12 $$ \frac{v_{\mathrm{dd}}-{v}_{\mathrm{A}}}{r_{\mathrm{ds},\mathrm{MP}1}}+{g}_{\mathrm{m},\mathrm{MP}1}{v}_{\mathrm{dd}}=\frac{v_{\mathrm{A}}}{r_{\mathrm{ds},\mathrm{MN}1}}+{g}_{\mathrm{m},\mathrm{MN}1}{v}_{\mathrm{A}} $$

From Eq. (12), the expression of the supply noise through path 3 to node A can be given by:

13 $$ {Av}_{\mathrm{path}3}=\frac{v_{\mathrm{A}}}{v_{\mathrm{dd}}}=\frac{r_{\mathrm{ds},\mathrm{MN}1}+{g}_{\mathrm{m},\mathrm{MP}1}{r}_{\mathrm{ds},\mathrm{MN}1}{r}_{\mathrm{ds},\mathrm{MP}1}}{r_{\mathrm{ds},\mathrm{MP}1}+{r}_{\mathrm{ds},\mathrm{MN}1}+{g}_{\mathrm{m},\mathrm{MN}1}{r}_{\mathrm{ds},\mathrm{MN}1}{r}_{\mathrm{ds},\mathrm{MP}1}} $$

The transconductance of the transistor operating in the subthreshold region is *g*_m_ = *I*_D_/*mV*_T_. Therefore, the relationship between *g*_m,MP1_ and *g*_m,MN1_ with the same current can be given as *g*_m,MP1_ = *g*_m,MN1_. Then, Eq. (13) can be simplified as:

14 $$ {Av}_{\mathrm{path}3}\approx 1 $$

Node B also has an effect on node A through path 1, but the effect is opposite to path 3, which can be expressed as:

15 $$ {Av}_{\mathrm{path}1}\approx -1 $$

For *V*_A_ = 2*V*_GS,MN2_, the gain of path 2 is given as:

16 $$ {Av}_{\mathrm{path}2}=-\frac{1}{2}{g}_{\mathrm{m},\mathrm{MN}2}\left(2{r}_{\mathrm{ds},\mathrm{MN}2}\Big\Vert \frac{1}{g_{\mathrm{m},\mathrm{MP}2}}\right)\approx -\frac{1}{2} $$

The effect of *v*_dd_ on node B through path 4 can be written as:

17 $$ {Av}_{\mathrm{path}4}=\frac{2{r}_{\mathrm{ds},\mathrm{MN}2}}{\left(1/{g}_{\mathrm{m},\mathrm{MP}2}\right)+2{r}_{\mathrm{ds},\mathrm{MN}2}}=\frac{2{g}_{\mathrm{m},\mathrm{MP}2}{r}_{\mathrm{ds},\mathrm{MN}2}}{1+2{g}_{\mathrm{m},\mathrm{MP}2}{r}_{\mathrm{ds},\mathrm{MN}2}} $$

From node A to node B in Fig. 2, two additional equations can be gotten, which are:

18 $$ {Av}_{\mathrm{path}4}{v}_{\mathrm{dd}}+{Av}_{\mathrm{path}2}{V}_{\mathrm{A}}={V}_{\mathrm{B}} $$

19 $$ {Av}_{\mathrm{path}3}{v}_{\mathrm{dd}}+{Av}_{\mathrm{path}1}{V}_{\mathrm{B}}={V}_{\mathrm{A}} $$

According to Eqs.(18) and (19), the noise at *V*_B_ can be given by:

20 $$ {V}_{\mathrm{B}}=\frac{2{g}_{\mathrm{m},\mathrm{MP}2}{r}_{\mathrm{ds},\mathrm{MN}2}-1}{1+2{g}_{\mathrm{m},\mathrm{MP}2}{r}_{\mathrm{ds},\mathrm{MN}2}}{v}_{\mathrm{dd}}\approx {v}_{\mathrm{dd}} $$

With the help of the proposed self-biased current source, the output node of the current generator part, B, can track the small-signal variation of the supply voltage, which is beneficial for the PSRR improvement of the whole voltage reference.

With a similar method, the supply noise gains of path 5 and path 6 can presented by Eqs. (21) and (22), respectively:

21 $$ {Av}_{\mathrm{path}5}={g}_{\mathrm{m},\mathrm{MP}3}\left({r}_{\mathrm{ds},\mathrm{MP}3}\Big\Vert \frac{1}{g_{\mathrm{m},\mathrm{MP}4}}\right) $$

22 $$ {Av}_{\mathrm{path}6}\approx 1 $$

Taken into consideration the noise path connection relationship of the reference generator shown in Fig. 2, the effect of the supply noise at the reference voltage, *V*_REF_, can be determined by path 5 and path 6:

23 $$ {v}_{\mathrm{REF}}={Av}_{\mathrm{path}5}{V}_{\mathrm{B}}+{Av}_{\mathrm{path}6}{v}_{\mathrm{dd}}=\frac{1}{1+{g}_{\mathrm{m},\mathrm{MP}4}{r}_{\mathrm{ds},\mathrm{MP}3}}{v}_{\mathrm{dd}}=\frac{1}{1+\frac{\exp \left({V}_{\mathrm{DS},\mathrm{MP}3}/{V}_{\mathrm{T}}\right)-1}{m}}{v}_{\mathrm{dd}} $$

For *V*_DS_ > 4*V*_T_, the exponential term in Eq. (23) is very large. This makes the PSRR performance to be greatly enhanced with *V*_DS,MP3_ increasing. In the proposed design, the minimum *V*_DS,MP3_ is over 200 mV, which means the change in the supply voltage has little effect on the *V*_REF_. Thus, the proposed structure has a good PSRR performance.

## Results and Discussion

**Fig. 4:**
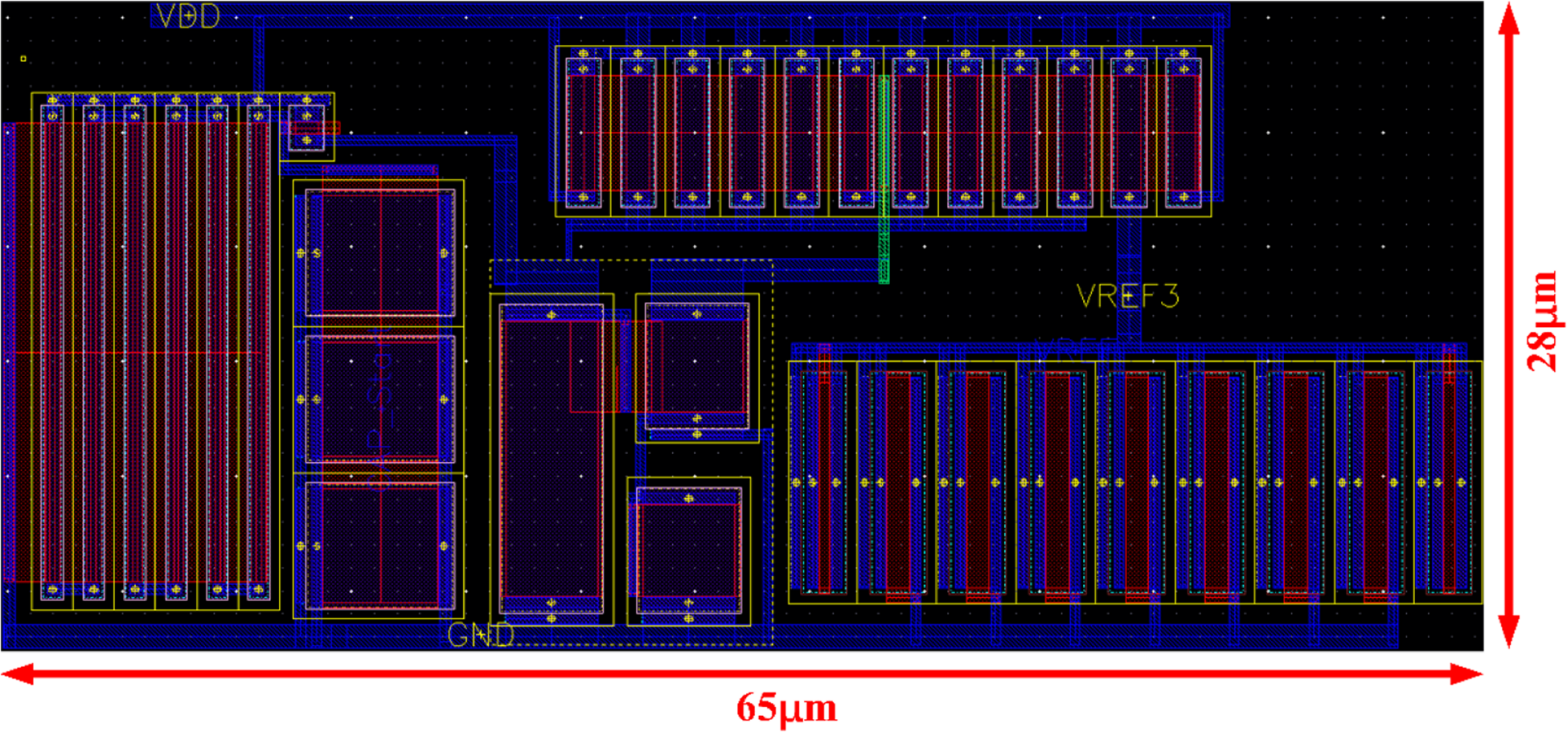
Layout of proposed circuit

The voltage reference is implemented in a 65-nm CMOS process, whose layout is shown in Fig. 4 occupying a 0.00182-mm^2^ active area.

**Fig. 5:**
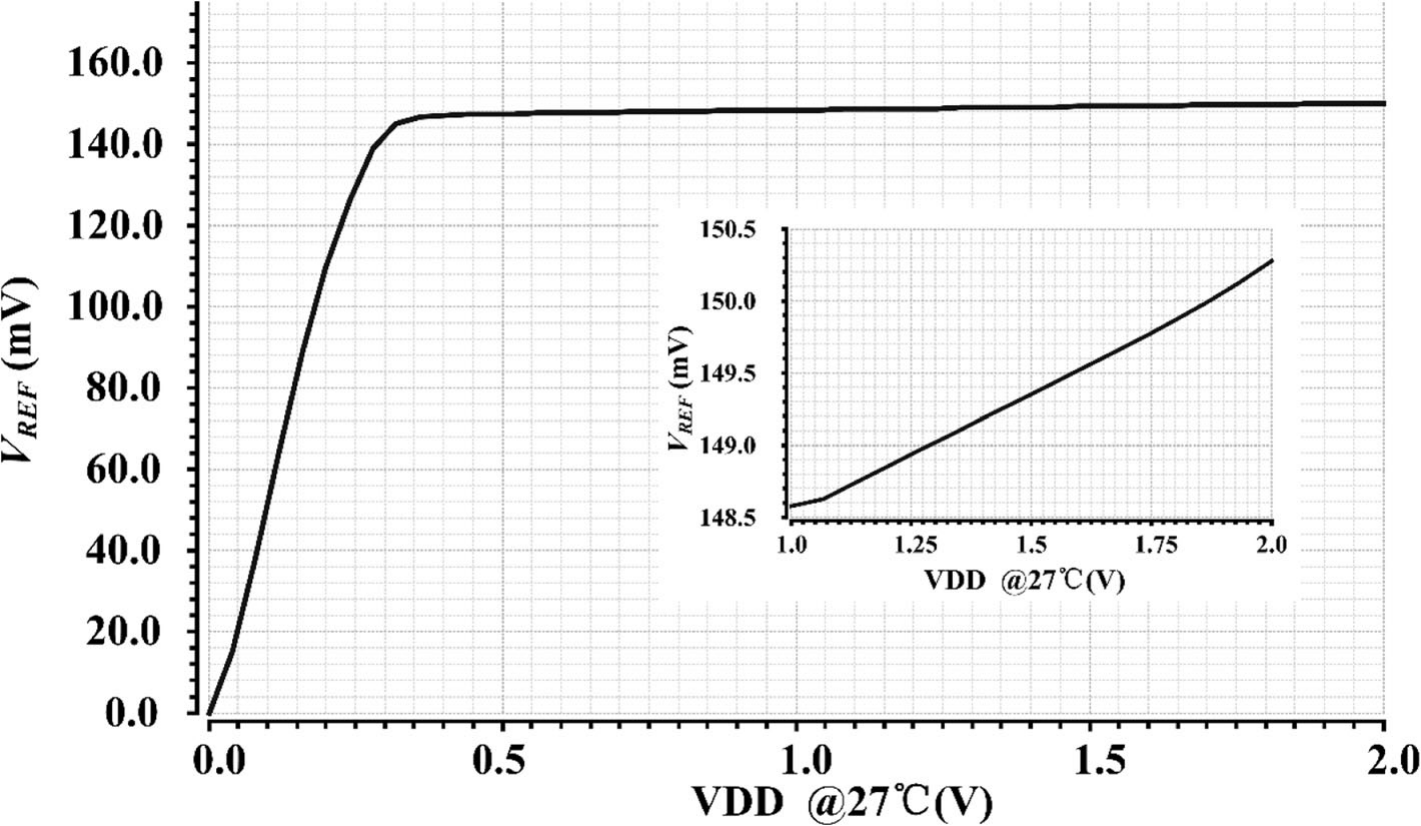
Waveform of *V*_REF_ versus supply voltage

Figure 5 shows the line regulation of the proposed voltage reference at 27 °C. As shown in Fig. 5, the minimum supply voltage can be as low as 350 mV, and the generated reference voltage, *V*_REF_, is around 148 mV*.* The line sensitivity (LS) is 1.8 mV/V.

**Fig. 6:**
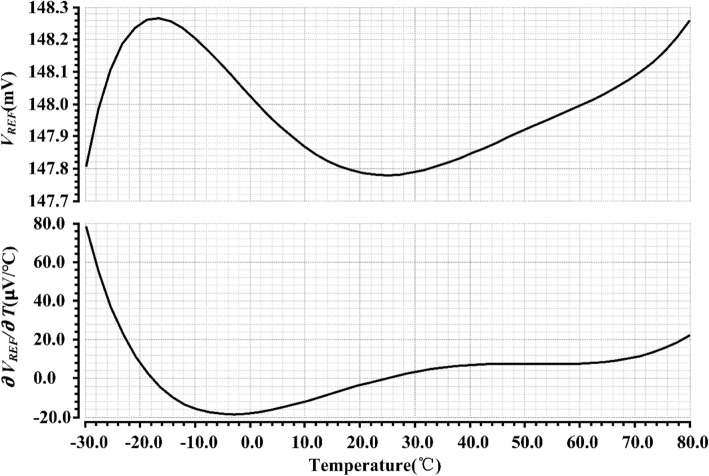
Temperature dependence of *V*_REF_

The temperature performance of *V*_REF_ with 350 mV supply voltage is shown in Fig. 6. The TC of *V*_REF_ is 28 ppm/°C from − 30 to 80 °C. *V*_REF_ shows positive temperature characteristics below − 15 °C and above 25 °C, while negative temperature characteristics at medium temperature region.

**Fig. 7:**
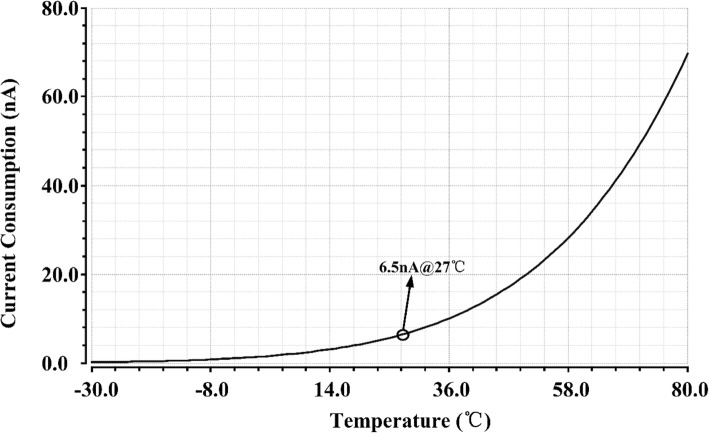
Current consumption versus temperature

Figure 7 shows the current consumption versus temperature with 350 mV supply voltage. The current shows a positive TC. The power consumption at room temperature is around 2.28 nW.

**Fig. 8:**
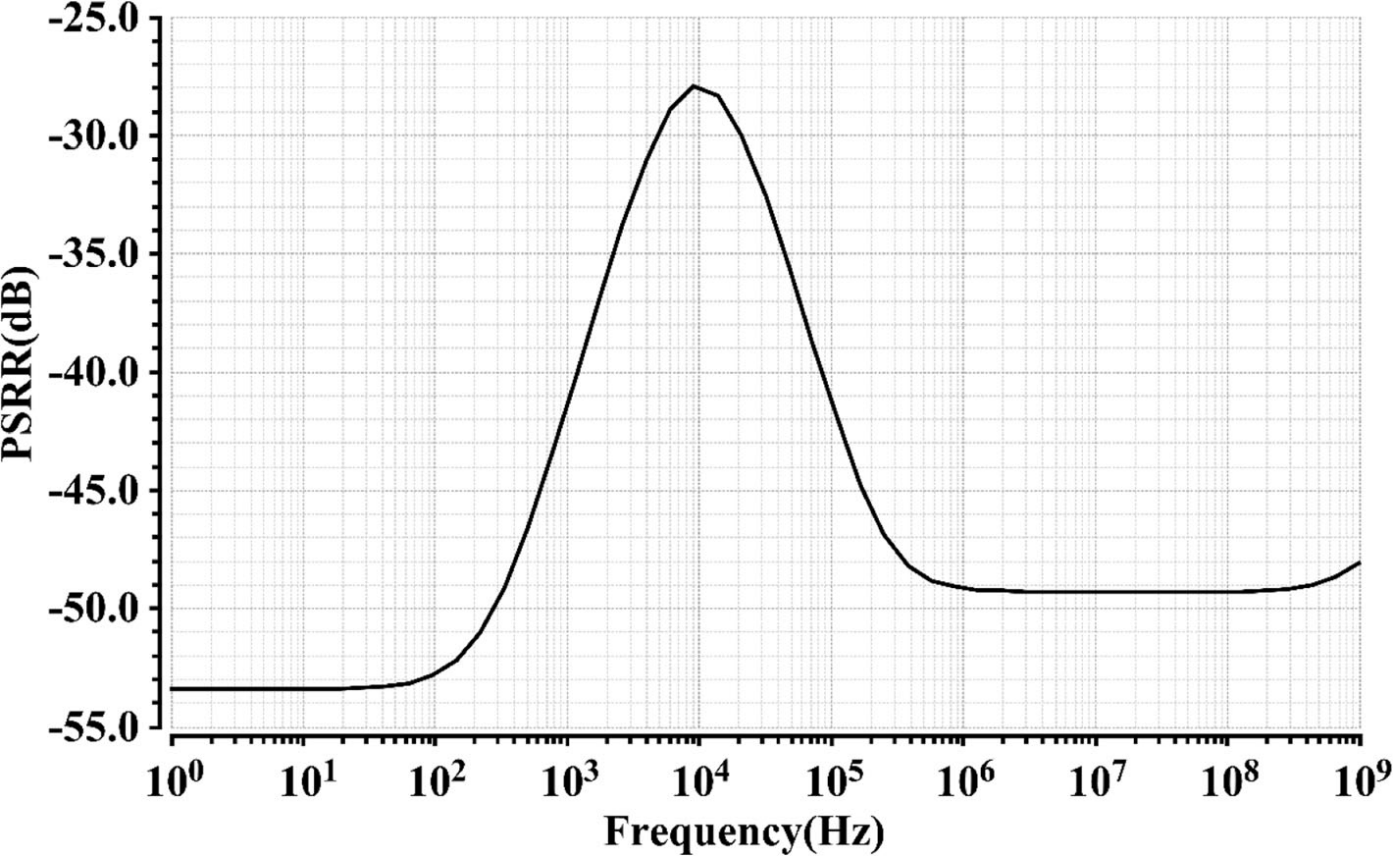
PSRR of proposed voltage reference

Figure 8 shows the result of PSRR at 27 °C with 350 mV supply voltage, where the PSRR without any output filter capacitor is over 53 dB up to 100 Hz. As mentioned above, the PSRR performance can be further improved with a supply voltage increase, which means the PSRR shown in Fig. 8 is the worst case of the proposed voltage reference.

**Fig. 9:**
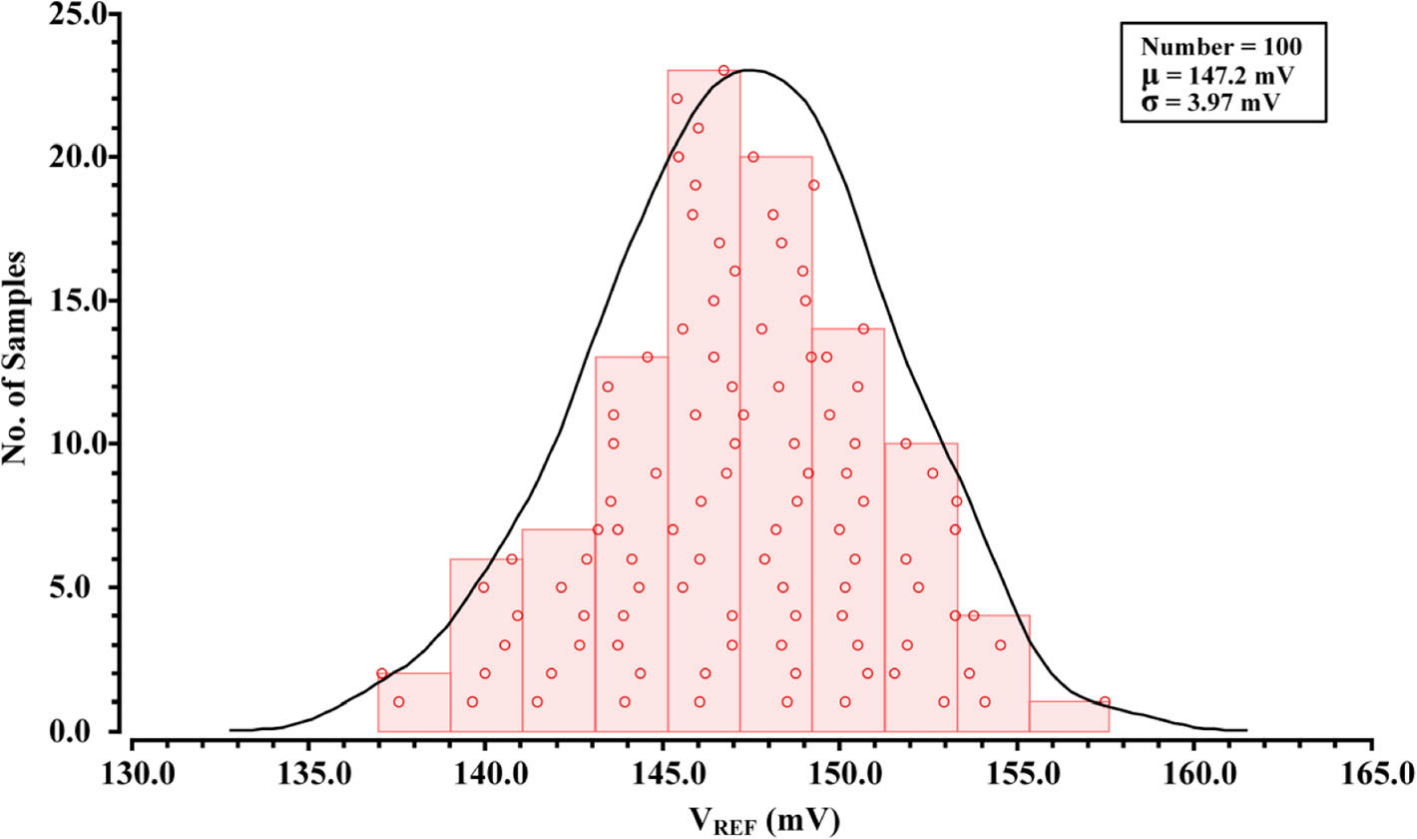
Distributions of untrimmed *V*_REF_

The distributions of untrimmed *V*_REF_ at 27 °C with 100 samples is shown in Fig. 9. The mean value and standard deviation of the *V*_REF_ is 147 mV and 3.97 mV, respectively, which results in a spread (σ/μ) of 2.7%.

Table 1 summarizes the characteristics of the proposed voltage reference and compares it with some previously reported voltage references.

**Table 1:** Performance summary and comparison

	This work	[14]	[8]	[9]
Process (nm)	65	130	180	65
Min. VDD (V)	0.35	0.5	1	0.2
Temp. range (°C)	− 30 to 80	− 40 to 120	− 40 to 125	− 40 to 85
*V*_REF_ (mV)	148	216	755	89.73
TC (ppm/°C)	28	192	49.6	790
Power (nW)	2.28	113	23	9.7
PSRR (dB) @100 Hz	− 53	− 27	− 52	− 42
LS (%/V)	1.2	13.7	0.524	2.01
Area (mm^2^)	0.00182	0.0016	0.0162	0.01

## Conclusion

A resistorless low-power voltage reference with high PSRR is presented in this paper, which is suitable for nanoscale applications and can be extended to more advanced process. With the help of self-biased current source based on MOSFET voltage divider, the required CTAT voltage, PTAT voltage, and weighted summation can be simultaneously realized in a compacted structure. What is more, a delta threshold voltage is chosen as the CTAT voltage, which has a greatly reduced negative TC. This also makes the required value of PTAT voltage to be shrunken. Therefore, the supply voltage and current consumption can be brought down. All the parts are only constructed by MOSFETs, which has priority in power-sensitive highly integrated applications, such as SOC.

## Abbreviations

BGR: Bandgap reference
CTAT: Complementary to absolute temperature
hvt: High threshold voltage
LS: Line sensitivity
mvt: Medium threshold voltage
PSRR: Power supply rejection ratio
PTAT: Proportional to absolute temperature
TC: Temperature coefficient
